# Active surveillance for thyroid Cancer: a qualitative study of barriers and facilitators to implementation

**DOI:** 10.1186/s12885-021-08230-8

**Published:** 2021-04-28

**Authors:** Catherine B. Jensen, Megan C. Saucke, Susan C. Pitt

**Affiliations:** 1 University of Wisconsin School of Medicine and Public Health, Madison, WI USA; 2 Wisconsin Surgical Outcomes Research Program, Department of Surgery, University of Wisconsin School of Medicine and Public Health, Madison, WI USA; 3 Division of Endocrine Surgery, Department of Surgery, University of Wisconsin School of Medicine and Public Health, 600 Highland Ave., CSC H4/724, Madison, WI 53792-7375 USA

**Keywords:** Thyroid cancer, Active surveillance, Implementation science, Qualitative, Barriers, Facilitators

## Abstract

**Background:**

The 2015 American Thyroid Association guidelines supported active surveillance (AS) as a strategy for managing select low-risk thyroid cancers. Data examining physicians’ attitudes about the acceptability of this option are limited. This study aimed to characterize the barriers and facilitators to implementing AS as perceived by practicing endocrinologists and surgeons in the United States.

**Methods:**

We conducted 24 semi-structured interviews probing physicians’ attitudes toward AS for patients with small, low-risk thyroid cancer. We used deductive content analysis guided by a well-known model of guideline implementation. Analysis characterized concepts and themes related to AS implementation as physician, guideline, or external factors. We performed member checking to validate results.

**Results:**

The most prominent barriers to AS were related to physician factors, although guideline-specific and external barriers were also observed. Physician attitudes towards AS comprised the majority of physician-related barriers, while lack of knowledge about the guideline was also discussed. Participants’ concerns about the potential negative outcomes resulting from observing a cancer were notable as were the lack of confidence in performing and offering surveillance. Beliefs about patient expectations and lack of knowledge about the guideline were also identified as barriers to offering surveillance. Guideline-specific and external barriers included the vagueness of surveillance protocols, lack of data supporting active surveillance, and societal beliefs about cancer. Facilitators of active surveillance included patients’ desire to avoid surgery and shared decision-making.

**Conclusions:**

Barriers and facilitators of active surveillance for low-risk thyroid cancers exist at multiple levels. Strategies to increase adoption of active surveillance should focus on physicians’ attitudes, patient expectations, data supporting surveillance outcomes, and promoting societal-level acceptance of surveillance.

## Background

Active surveillance of low-risk thyroid cancer is a newer alternative approach to immediate surgery that has not been widely accepted in the United States (US). In 2015, the American Thyroid Association (ATA) guidelines supported active surveillance for select patients, such as those with ≤1 cm papillary thyroid cancer [1]. This change was based on data from Japan showing low rates of disease progression while surveilling these small cancers [2, 3]. Since the guideline change, studies from other countries have also supported this approach [4–7]. While prospective trials are underway in the United States (US) [8, 9], data remain limited, and as with any new approach, barriers to implementation exist [10–12].

This qualitative study was designed to take an in-depth examination of barriers and facilitators to active surveillance in the US. Current data on physicians’ attitudes towards active surveillance in the US are primarily based on surveys and have not to the best of our knowledge employed a qualitative approach [13–15]. A clear, comprehensive understanding of barriers to active surveillance is necessary for developing and implementing effective strategies that increase use of this approach. Because overtreatment of patients with small, low-risk thyroid cancer remains a significant concern, increased use of active surveillance has the potential to reduce the resulting morbidity [16–19].

## Methods

### Study population and recruitment

To characterize the barriers and facilitators of active surveillance for low-risk thyroid cancer, we conducted semi-structured interviews with actively practicing thyroid surgeons and endocrinologists in the US. Participants were identified and recruited at the 86th Annual ATA meeting as previously described [16]. The Institutional Review Board approved this study (No. 2016–0884). Participants provided written informed consent to participate in this study which included the publication of anonymized direct quotations from interviews for publication.

### Data collection

Interviews were performed by two researchers with advanced training in qualitative interviewing (MCS, EMW). The interview guide was developed in conjunction with qualitative research experts and key stakeholders, including the target audience and thyroid cancer survivors, and piloted. The guide included a case-based clinical vignette and open-ended questions such as:

- What is your response to the ATA guideline that endorses active surveillance?
- What is your current approach to active surveillance?
- What concerns do you have about active surveillance?
- How did you implement active surveillance in your practice?

### Analysis

Interviews were audio-recorded, transcribed, and deidentified. NVivo 11 (QSR International) was used for data management. To create the initial codebook, interview transcripts (*n* = 3) were analyzed using open coding by a team with diverse backgrounds in sociology (MCS), thyroid surgery (SCP), and population health (JLJ). As coding continued, emergent themes were incorporated, and taxonomy revised using constant comparative analysis. We resolved discrepancies between coders through discussion and group consensus.

**Fig. 1:**
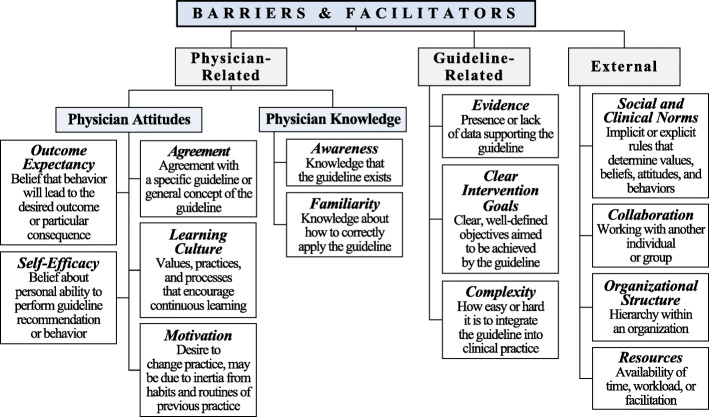
Framework of guideline implementation developed by Fischer et al. categorizes barriers and facilitators to into three domains—physician-related, guideline-related, and external factors. Each barrier/facilitator and its definition are shown here

Higher-level analysis of barriers and facilitators involved a deductive strategy to characterize and map all codes and themes onto Fischer’s Guideline Implementation Framework [20]. We selected this framework because it builds on Cabana’s well-known model and identifies strategies to address each barrier [21]. Fischer’s framework differentiates barriers and facilitators into three levels: physician-related (personal), guideline-related, and external factors (Fig. 1).

### Member checking

Because initial data collection occurred within a year of the ATA guidelines release, we performed member checking in August–September 2020 to ensure the credibility and applicability of results [22]. We emailed all participants a detailed summary of the results and asked about current relevance and emergence of new barriers or facilitators. Sixteen responded; all but one confirmed that the results resonated with their experiences and no new barriers or facilitators had developed in their practice since the initial interviews. Appropriate revisions were made based on the feedback.

## Results

Twenty-four physicians including 12 surgeons and 12 endocrinologists participated in semi-structured interviews about active surveillance of low-risk thyroid cancer. Participants had a median age of 43 years (range 34–68) (Table 1). The majority were male (71%), white (88%), and in academic practice (88%). Most participants (67%) saw at least 10 patients/year with ≤1 cm thyroid cancers. To protect anonymity, quotes are identified as “surgeon” or “endocrinologist.”

**Table 1:** Demographic data

	*Surgeons* n (%)	*Endocrinologists* n (%*)*
Age (median, range)	41 (34–67)	47 (35–68)
Female	2 (18)	5 (42)
Caucasian	11 (92)	10 (83)
Academic practice	11 (92)	10 (83)
Location		
East/Northeast	4 (36)	6 (42)
South	2 (18)	0 (0)
Midwest	2 (18)	4 (33)
West	2 (18)	1 (8)
Treat > 10 PTMC/year	8 (67)	8 (67)
> 50% of practice is thyroid cancer	12 (100)	6 (50)
> 25 thyroid surgeries/year	12 (100)	–
Member of the ATA	12 (100)	12 (100)
Read all of 2015 ATA guidelines	7 (58)	9 (75)
Read at least part of 2015 ATA guidelines	4 (33)	2 (17)

*PTMC* Papillary microcarcinoma (defined as papillary thyroid cancer measuring 1 cm or smaller); *ATA* American Thyroid Association

### Barriers to active surveillance

#### Physician-related factors

Several key barriers to active surveillance emerged at the physician-level, including physician attitudes and knowledge.

##### Physician attitudes

***Outcome Expectancy:*** The most prominent barriers discussed during interviews were related to participants’ beliefs about anticipated patient- and disease-related outcomes or consequences of active surveillance. Participants frequently expressed concern about the risk of metastasis if cancer remained in place. For example, Surgeon 5 stated, “*For my own personal beliefs and biases, I feel that any potential risk for cancer to develop and spread throughout the body is unacceptable*.” Participants also anticipated experiencing negative emotions, such as guilt, if an adverse outcome occurred during surveillance. Surgeon 1 described, “*I would feel uncomfortable … if it grew or spread then I feel like that would be on my shoulders*.”

In addition, almost half of participants expressed concern that adverse patient outcomes could result from loss of follow-up. They described hesitancy to offer surveillance to unreliable patients because of the potential for cancer progression. For example, Surgeon 6 explained, “*it would be safer to* [remove] *half the thyroid if the patient is never going to come back.*” Regardless of specialty, participants often viewed surgery as “*definitive therapy*” (Endocrinologist 10) that provided “*certainty*” (Endocrinologist 1) and a predictable, expected outcome, whereas surveillance introduced uncertainty.

Some participants also worried that patients would file lawsuits if they experienced an adverse outcome during surveillance. They believed it was important to disclose the potential for poor outcomes prior to initiating surveillance. Surgeon 10 described feeling “*legally obligated to say, ‘Waiting can allow that cancer a chance to progress.’*”

While many participants described concern about cancer spreading, others acknowledged that metastasis rarely occurs during surveillance. For example, Surgeon 8 stated, “*every clinician is afraid they’re gonna be surveilling somebody who explodes with lymph node disease … the truth of the matter is that’s extremely unlikely to ever happen*.”

**Fig. 2:**
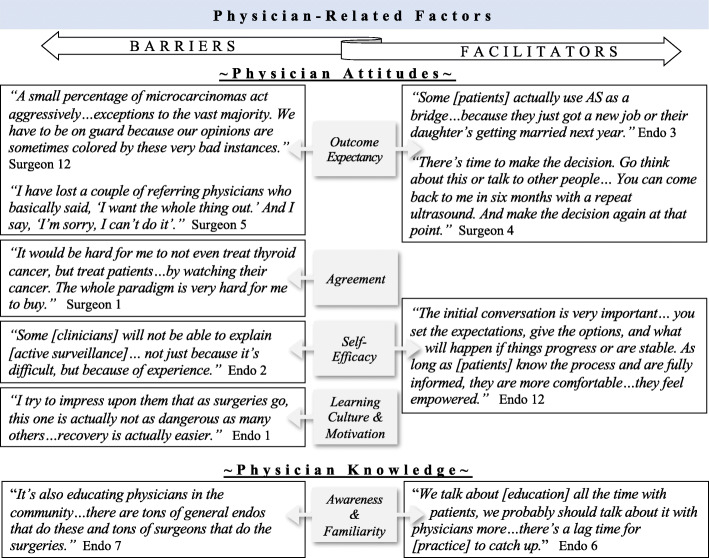
Exemplary quotes of physician-related factors as barriers and facilitators to implementing active surveillance (AS) of low-risk thyroid cancer. (Abbreviation: Endo, endocrinologist)

In addition to concern about outcomes, some participants discussed the impact of surveillance on their practice and potential for losing patients and/or referring providers as consequence of not offering thyroidectomy (Fig. 2). Even non-surgeons acknowledged patients would seek second opinions and eventually find “*a surgeon who would agree to do* [surgery]” (Endocrinologist 1).

***Agreement:*** A less prominent barrier to active surveillance was lack of agreement with the guidelines and concern about the concept of watchful waiting. Some participants described being hesitant to recommend surveillance because they are “*not treating*” (Fig. 2) and patients might “*feel like we’re not caring for them”* (Surgeon 6).

***Self-Efficacy:*** Participants also discussed lacking confidence in performing active surveillance. Those who viewed their own lack of experience as a barrier to discussing and offering the approach tended to describe surveillance in vague, as opposed to detailed, terms (Fig. 2). Participants also mentioned their inability to convince patients to undergo surveillance because the option is “*hard to sell*” (Surgeon 1).

***Learning Culture*** & ***Motivation:*** While no participants were unwilling to implement active surveillance in their own practice, many expressed doubts about other physicians’ motivation to learn about and incorporate surveillance clinically. Participants believed other physicians are aware of the guidelines, but choose not to follow them or present surveillance as an option (Fig. 2). Specifically, some participants thought surgeons were not open to surveillance because “*they are going to want to cut*” (Endocrinologist 6). Meanwhile, surgeons often described thyroidectomy as a “*low-risk*” option that “*provides good results*” (Surgeons 7 and 8).

##### Physician knowledge

***Awareness*** *&* ***Familiarity:*** Few participants expressed concern regarding complete lack of awareness of the guidelines supporting active surveillance. However, many believed lack of familiarity with and/or inappropriate application of the guidelines were barriers. Participants described lag time from guideline release to clinical implementation and expressed that education on how to perform surveillance is lacking (Fig. 2).

#### Guideline-related factors

Barriers to active surveillance that emerged at the guideline-level included lack of evidence, lack of clear intervention goals, and complexity.

##### Evidence

**Fig. 3:**
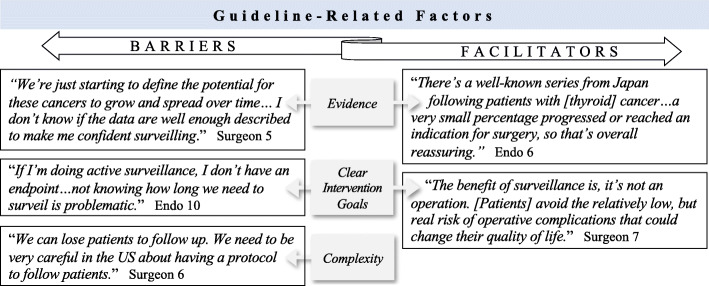
Exemplary quotes of guideline-related factors as barriers and facilitators to active surveillance of low-risk thyroid cancer. (Abbreviation: Endo, endocrinologist)

The most apparent guideline-related barrier was concern about the lack of evidence supporting active surveillance. Many participants believed that data were insufficient and highlighted the lack of prospective trials of patients undergoing surveillance in the US (Fig. 3). While some participants discussed trials conducted in Japan as support for active surveillance, several others expressed hesitancy as this data “*may not apply to the US population*” (Endocrinologist 2).

##### Intervention goals

Many participants believed the guidelines lacked clear recommendations and expressed concerns about the goals and duration of active surveillance. Participants described a desire for clear surveillance protocols with more explicit details about patient follow-up, age-specific recommendations, and universal thresholds for nodule progression that prompt further intervention (Fig. 3).

##### Complexity

Participants also discussed that the written guidelines for active surveillance were vague and cited the difficulty and risks of implementing surveillance in their practice without a clear system of patient follow-up in place (Fig. 3).

#### External factors

Barriers to active surveillance on the external-level focused on social and clinical norms, lack of collaboration, organizational constraints, and lack of resources.

##### Norms

**Fig. 4:**
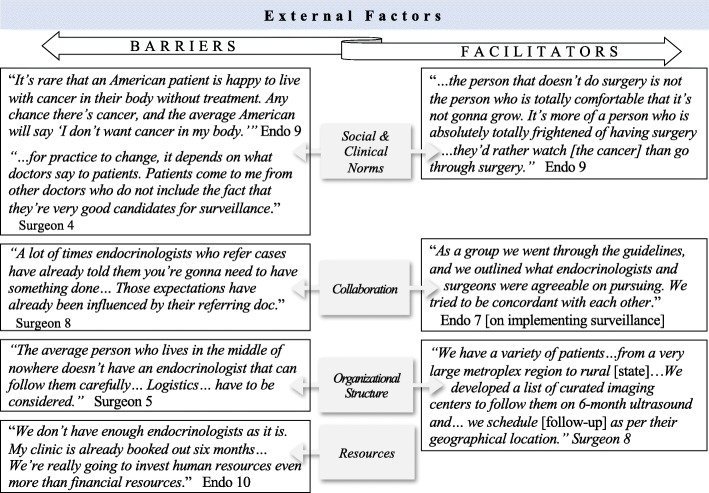
Exemplary quotes of external factors as barriers and facilitators to active surveillance of low-risk thyroid cancer. (Abbreviation: Endo, endocrinologist)

Barriers related to social and clinical norms were prevalent throughout the interviews, particularly concerns over societal attitudes and beliefs about cancer. All participants discussed the impact of “*the C word*” on patients’ willingness to consider active surveillance (Fig. 4). Most described this barrier as resulting from “*being in a culture where cancer is bad and needs to get out*” (Surgeon 1).

Participants discussed the influence of clinical norms on patient expectations. Surgeons in particular described how once a patient is referred to a surgeon, it is difficult to get the patient to consider non-surgical management (Fig. 4). For example, Surgeon 6 stated, “*It’ll take a long time for active surveillance to be put into practice from the surgeon’s perspective … because* [patients] *expect surgery when they come to see you*.”

##### Collaboration

Participants believed lack of collaboration between specialists poses a barrier to active surveillance. They highlighted the potential for patients to receive conflicting advice about treatment options (Fig. 4). Some participants discussed how patient expectations set by referring providers can “*create lots of conflict*” if their own recommendation does not align with that of the referring provider (Surgeon 3).

##### Organizational constraints

Participants also discussed that active surveillance may be feasible on the individual provider-level, but most participants saw their ability to implement surveillance as being limited by lack of organizational infrastructure and their larger practice environment (Fig. 4). Organization-level implementation would require “*institution-specific protocols for monitoring patients*” which can be difficult to put in place (Endocrinologist 5).

##### Resources

Participants additionally identified numerous resource-related barriers to active surveillance. These barriers included the lack of patient access to ongoing care, lack of skilled community ultrasound technologists, and the increased physician workload that would result from surveilling a large cohort of patients (Fig. 4).

### Facilitators of active surveillance

While multiple barriers to active surveillance emerged in the interviews, several facilitators were also identified.

#### Physician-related factors

Physician-related facilitators of active surveillance included positive attitudes about outcome expectancy and self-efficacy as well as knowledge, awareness, and familiarity with the guidelines (Fig. 2). Some participants supported the open-ended structure of active surveillance and framed surveillance as a “*bridge*” to future, more definitive treatment. Most participants identified shared decision-making as another facilitator of surveillance (Fig. 2). In terms of physician knowledge, participants described the role of physician education in improving guideline awareness and familiarity and saw continuing medical education as a strategy to increase knowledge about how to perform active surveillance (Fig. 2).

#### Guideline-related factors

At the guideline-level, clear intervention goals was the only facilitator identified. Nearly all participants described how the goal of the guideline was to avoid surgery and its potential complications, which was a benefit and facilitator of active surveillance (Fig. 3).

#### External factors

Facilitators of active surveillance that emerged at the external-level included social and clinical norms, particularly related to attitudes and beliefs about surgery and the presence of collaboration (Fig. 4). Many participants described patients’ fear of surgery as a facilitator, citing how surveillance is often the treatment choice for patients who are more afraid of surgery than the possibility of cancer progression. Most participants also expressed that collaboration and a multidisciplinary team approach facilitates guideline implementation (Fig. 4).

## Discussion

This qualitative study characterized barriers and facilitators to implementing active surveillance for patients with small, low-risk thyroid cancer in the United States (US) [20]. The most prominent barriers were physician-related. Physicians’ beliefs about fear about disease progression and their own ability to offer and perform surveillance contributed to a majority of perceived barriers, while knowledge about guidelines contributed to a lesser extent. Participants also viewed vagueness of surveillance protocols and limited long-term outcome data supporting active surveillance in the US as impeding implementation. Patient expectations and societal beliefs about cancer were also commonly described barriers to implementation. Interventions to address some of these barriers are already underway, including establishing outcomes of active surveillance in US patients [8, 9], publication of protocols [23–27], and physician education. Additional interventions should focus on physicians’ and patients’ beliefs about cancer, facilitating multidisciplinary approaches, and promoting societal-level acceptance of active surveillance.

Physicians’ beliefs about the potential negative outcomes of active surveillance was one of the most significant barriers identified. Fear of cancer metastasis and losing patients to follow-up during surveillance appear to hinder physicians from offering surveillance as an option. These fears are reinforced by anecdotal experience and patient expectations about surgery. To effect behavioral change going forward, it will be important to transform physicians’ beliefs about the negative consequences of surveillance. Potential interventions include audit-feedback systems, disseminating surveillance protocols, and publishing the data currently being collected on oncologic and patient-reported outcomes. Individualized audit-feedback on patient care decisions has been shown to be effective in other diseases [28, 29]. Multispecialty local- or state-level efforts that incorporate peer coaching may be an alternative strategy to audit-feedback. In other medical settings, coaching has been shown to improve ongoing skills and optimize patient outcomes [30, 31]. These methods could be further supported with educational webinars by national organizations.

Physicians’ self-efficacy and beliefs about their ability to offer or perform active surveillance were also prominent barriers. Participants discussed lack of training and proficiency in shared decision-making, limited experience performing surveillance, and being uncomfortable discussing surveillance. Interventions to promote physician confidence include individualized or group training, education, clinical decision-making support tools, and reminders that prompt physicians to consider active surveillance [24, 32]. Recent studies in Canada and the US showed that patients would consider less extensive management options if recommended by their physician [16, 33–35]. Therefore, physicians must become comfortable discussing and offering active surveillance as an option. Patient-focused education may also play a role; increasing patients’ knowledge and promoting discussion about surveillance could further support acceptance and implementation of this approach [36–38].

While interventions targeted at physicians and patients are important, overarching societal beliefs must also be addressed. We found that the culturally rooted belief that “you cannot watch cancer” poses a substantial, but not insurmountable, barrier to active surveillance. In the US, active surveillance of low-risk prostate cancer has become an acceptable alternative to active treatment and trials are assessing this approach in women with ductal carcinoma in situ [39]. Learning from prostate cancer implementation efforts may accelerate acceptance of active surveillance for thyroid cancer [40]. Strategies to promote societal-level change include national campaigns raising awareness about low-risk thyroid cancer, publishing longitudinal cohort and randomized studies establishing active surveillance safety and outcomes, and developing robust social support networks for patients undergoing surveillance [41].

Clinical norms related to patient and referring provider expectations of surgical management also pose a barrier to active surveillance. Potentially feasible strategies to reset expectations include local adaptation of guidelines with multidisciplinary collaboration, outreach to community providers, and reframing treatment recommendations. Local adaptation supported by stakeholder groups and task forces enables incorporation of active surveillance into established clinical structures across specialties [42]. Multidisciplinary collaboration further facilitates acceptance and reduces specialty-specific bias when considering management options [43].

While this study identified several key barriers and facilitators of active surveillance, it has limitations. First, while we conducted interviews at a national specialty meeting to facilitate physician sample heterogeneity with respect to geography, background, and training, participants’ level of knowledge and experience with active surveillance was relatively high compared to most US physicians who treat thyroid cancer. The data were also collected within a year of ATA guideline release. It is possible that some barriers have emerged, changed, or resolved since the data were collected. To address this limitation and ensure the reliability and applicability of our data, we recently performed formal member checking to confirm that the barriers and facilitators described remain relevant and no new barriers have arisen. However, additional barriers may still exist. New data on patient outcomes and protocols for active surveillance have also been published in the interim [4–6, 23–26]. Nonetheless, this study is the first we are aware of to qualitatively highlight the role of physician attitudes and beliefs in adoption of active surveillance in the US.

## Conclusion

This in-depth, qualitative study identified barriers and facilitators of active surveillance of thyroid cancer in the US as perceived by practicing thyroid surgeons and endocrinologists. The most prominent barriers included physician attitudes as well as societal and clinical norms. The findings indicate that adoption of active surveillance can be increased by provider-, patient-, system-, and societal-level interventions. These interventions should address physicians’ attitudes and beliefs about the outcomes of surveillance, increase physicians’ confidence performing surveillance, and promote societal acceptance of surveillance as a management option.

## Data Availability

The datasets generated and/or analysed during the current study are not publicly available due to ethical reasons, as they contain information that could compromise the privacy and consent of research participants but are available from the corresponding author (SCP) on reasonable request.

## Abbreviations

ATA: American thyroid association
US: United States
